# Multiple evidences reveal new species and a new record of smelly *Gymnopus* (Agaricales, Omphalotaceae) from China

**DOI:** 10.3389/fmicb.2022.968617

**Published:** 2022-10-06

**Authors:** Jia-Jun Hu, Li-Ru Song, Yong-Lan Tuo, Gui-Ping Zhao, Lei Yue, Bo Zhang, Yu Li

**Affiliations:** ^1^Engineering Research Center of Edible and Medicinal Fungi, Ministry of Education, Jilin Agricultural University, Changchun, China; ^2^School of Life Science, Northeast Normal University, Changchun, China; ^3^China Mycological Valley (Hefei), Hefei, China; ^4^Gansu Engineering Laboratory of Applied Mycology, Hexi University, Zhangye, China; ^5^Joint Laboratory of International Cooperation in Modern Agricultural Technology, Ministry of Education, Jilin Agricultural University, Changchun, China

**Keywords:** foetid smell, *Gymnopus* sect. *Impudicae*, new species, phylogenetic analysis, taxonomy

## Abstract

*Gymnopus* sect. *Impudicae* is a poorly studied group around the world. However, it is well known for its pungent smell—a total of five species from China belonging to sect. *Impudicae* were recorded, and included four species new to science—*G. epiphyllus, G. cystidiosus, G. subdensilamellatus*, and *G. subpolyphyllus*—which were delimited and proposed based on morphological and molecular evidences, and one new record from Henan, Jiangxi, and Gansu Province, China—*G. densilamellatus*. Detailed descriptions and illustrations were presented as well as comparisons to similar species. Phylogenetic analysis inferred from the ITS and nLSU dataset supported the *Gymnopus* as a monophyletic genus which was defined by Oliveira et al., and the novel species grouped as separate lineages within it. A Key to the reported species of *Gymnopus* sect. *Impudicae* is also provided.

## Introduction

*Gymnopus* (Pers.) Roussel (Omphalotaceae, Agaricales) is a widely spread and controversial genus. In the conception of Antonín and Noordeloos (2010), *Gymnopus* was composed of sect. *Impudicae* (Antonín and Noordel.) Antonín and Noordel., sect. *Androsacei* (Kühner) Antonín and Noordel., sect. *Levipedes* (Quél.) Halling, and sect. *Vestipedes* (Fr.) Antonín, Halling, and Noordel. Later, the definition of *Gymnopus* is modified constantly. Sect. *Perforanita* (Singer) R.H. Petersen was combined into *Gymnopus* (Petersen and Hughes, 2016). Recently, Oliveira et al. (2019) redefined the genus *Gymnopus* more strictly based on combined ITS + nLSU phylogenetic analysis. In their conception, the key features of the genus *Gymnopus* including collybioid basidiomata, rarely tricholomatoid or marasmioid, free, emarginate or adnate lamellae that are usually crowded, and insititious stipe or not, usually with a strigose base, a white spore print; basidiospores ellipsoid to short-oblong, inamyloid; cheilocystidia usually present and variety; a cutis or ixocutis pileipellis with radially arranged cylindrical hyphae or interwoven more like a trichoderm or ixotrichoderm, made up of irregularly coralloid terminal elements (‘*Dryophila* structures')—often incrusted, diverticulate hyphal elements, mixed with broom cells and coralloid hyphae; and clamp connections present in all tissues. Therefore, sect. *Vestipedes* was segregated and placed within *Collybiopsis* (J. Schröt.) Earle (=*Marasmiellus* Murrill), and sect. *Perforanita* was proposed as a new genus—*Paragymnopus* J.S. Oliveira. In addition, some *Gymnopus* species were transferred to two new genera—*Paramycetinis* R.H. Petersen and *Pseudomarasmius* (Petersen and Hughes, 2020).

*Gymnopus* sect. *Impudicae* was treated as a subsection of sect. *Vestipedes* initially, then Antonín and Noordeloos (2010) raised it to a section rank based on the morphology and molecular evidence. Additionally, some previously assigned species as *Micromphale* Gray, *Marasmiellus* sect. *Gloeonemae* (Kühner) Antonín and Noordel., and *Marasmius* sect. *Gloeonemae* Kühner was transferred to this section. Sect. *Impudicae* is characterized by most species with a strong and usually unpleasant smell, reminding of rotten cabbage or onion, pileipellis with diverticulate terminal elements, and typically inconspicuous cheilocystidia (Antonín and Noordeloos, 1993, 1997; Antonín and Noordeloos, 2010). Until now, about 300 species belonging to *Gymnopus s. str*. were described worldwide, and 26 species were included in sect. *Impudicae* (Kirk et al., 2008). In China, the species diversity, taxonomy, and phylogeny of macrofungi have been investigated in recent years, thus many new species have been discovered (Zhong et al., 2018; Cui et al., 2019; Sun et al., 2020, 2022; Zhang et al., 2020; Cao et al., 2021a,b; Hu et al., 2021b; Liu et al., 2021, 2022a,b; Ji et al., 2022; Tuo et al., 2022; Wang et al., 2022). However, research on sect. *Impidicae* in China lags behind the studies in Europe, the United States, or South Korea (Ryoo et al., 2016) and lacks systematic analysis. Only eight species were recorded from China in sect. *Impidicae* (Li et al., 2021a). Furthermore, only *G. densilamellatus* Antonín, Ryoo, and Ka (Li et al., 2021a) *, G. foetidus* (Sowerby) J.L. Mata and R.H. Petersen (Deng, 2016), and *G. polyphyllus* (Peck) Halling (Ding, 2017) were recorded from northern China.

*Gymnopus* species are found to have economic value: for example, *G. erythropus* (Pers.) Antonín, Halling and Noordel. and *G. ocior* (Pers.) Antonín & Noordel. are edible macrofungi (Wu et al., 2019). However, some species belongs this genus were found to be poisonous fungi, e.g., *G. densilamellatus* was also discovered from Hunan, Guizhou, and Hebei Province, and resulted in several poisoning incidents with gastroenteritis symptoms (Wu et al., 2019; Li et al., 2021c, 2022).

This paper aims to describe and illustrate five species of *Gymnopus* sect. *Impudicae*—four species new to science and one new record for Henan, Jiangxi, and Gansu Province, China—based on morphology and molecular evidence.

## Materials and methods

### Sampling and morphological studies

The studied specimens were photographed *in situ*. The size of the basidiomata was measured when fresh. After examining and recording the fresh macroscopic characters, the specimens were dried in an electric drier at 40–45°C.

The macroscopic characteristics were based on field notes and photographs, with the color descriptions corresponding to the Flora of British Fungi: color identification chart (Royal Botanic Garden, 1969). The dried specimens were rehydrated in 94% ethanol for microscopic examination. And then mounted in 3% potassium hydroxide (KOH), 1% Congo red solution (0.1 g Congo red dissolved in 10 mL distilled water), and Melzer's reagent (1.5 g potassium iodide, 0.5 g crystalline iodine, and 22 g chloral hydrate dissolved in 20 mL distilled water) (César et al., 2018); they were then examined with a Zeiss Axio lab. A1 microscope at magnifications up to 1,000×. All measurements were taken from the sections mounted in the 1% Congo red. For each specimen, a minimum of 40 basidiospores, 20 basidia, 20 cheilocystidia, and 20 hyphal elements of pileipellis were measured from two different basidiocarps. When reporting the variation in the size of the basidiospores, basidia, cheilocystidia, and hyphal elements of the pileipellis, 5% of the measurements were excluded from each end of the range and are given in parentheses. The basidiospore measurements are length × width (L × W). Q denotes the variation in the ratio of L to W among the studied specimens, Qm denotes the average Q value of all the basidiospores ± standard deviation. The specimens examined are deposited in the Herbarium of Mycology of Jilin Agricultural University (HMJAU).

### DNA extraction, PCR amplification, and sequencing

According to the manufacturer's instructions, the total DNA was extracted from dried specimens using the NuClean Plant Genomic DNA Kit (Kangwei Century Biotechnology Company Limited, Beijing, China). Sequences of the internal transcribed spacer (ITS) region, and nuclear large ribosomal subunits (nLSU) were used for phylogenetic analysis. The ITS sequence was amplified using the primer pair ITS1-F (CTT GGT CAT TTA GAG GAA GTA A) and ITS4-B (CAG GAG ACT TGT ACA CGG TCC AG) (Gardes and Bruns, 1993). The nLSU sequence was amplified using the primer pair LR0R (GTA CCC GCT GAA CTT AAG C) and LR7 (TAC TAC CAC CAA GAT CT) (Vilgalys and Hester, 1990; Cubeta et al., 1991). PCR reactions (25 μL) contained 8 μL 2 × EasyTaq^®^ PCR SuperMix (TransGen Biotech Co., Ltd., Beijing, China), 1 μL 10 μM primer L, 1 μL 10 μM primer R, 3 μL DNA solution, and 12 μL dd H_2_O (Hu et al., 2021a, 2022). The reaction programs were as follows: for ITS, initial denaturation at 94°C for 4 min, followed by 30 cycles at 94°C for 1 min, 54°C for 1 min and 72°C for 1 min, and a final extension of 72°C for 10 min (Coimbra et al., 2015); for nLSU, initial denaturation at 95°C for 3 min, followed by 30 cycles at 94°C for 30 s, 47°C for 45 s, and 72°C for 90 s, and a final extension of 72°C for 10 min (Ryoo et al., 2016; Hu et al., 2021b). The PCR products were visualized *via* UV light after electrophoresis on 1% agarose gels stained with ethidium bromide and purified using the Genview High-Efficiency Agarose Gels DNA Purification Kit (Gen-View Scientific Inc., Galveston, TX, USA). The purified PCR products were then sent to Sangon Biotech Limited Company (Shanghai, China) for sequencing using the Sanger method. The new sequences were deposited in GenBank (http://www.ncbi.nlm.nih.gov/genbank; Table 1).

**Table 1:** Voucher/specimen numbers, Country, and GenBank accession numbers for the specimens of *Gymnopus* and related genera used in the study; sequences produced in this study are bold.

**Scientific name**	**Country**	**Voucher/specimen numbers**	**GenBank accession numbers**		**Reference**
			**ITS**	**LSU**	
*Gymnopus alliifoetidissimus*	China	GDGM76695	MT023344	MT017526	Li et al., 2021a
*G. androsaceus*	France	CBS239.53	MH857174	MH868713	Vu et al., 2019
*G. androsaceus*	Russia	TENN-F-59594	KY026663	KY026663	Petersen and Hughes, 2016
*G. atlanticus*	Brazil	URM87728	KT222654	KY302698	Coimbra et al., 2015
*G. barbipes*	USA	TENN67858	KJ416269	KY019642	Petersen and Hughes, 2014
*G. brassicolens*	Russia	TENN55550	DQ449989		Mata et al., 2006
*G. ceraceicola*	New Zealand	PDD87181	KC248405		Cooper and Leonard, 2013
***G. cystidiosus***	**China**	**HMJAU60992**	**ON259024**	**ON259036**	**This study**
***G. cystidiosus***	**China**	**HMJAU60993**	**ON259025**	**ON259037**	**This study**
***G. cystidiosus***	**China**	**HMJAU60994**	**ON259026**	**ON259035**	**This study**
*G. densilamellatus*	China	HMJAU49128	MT023351	MT017529	Li et al., 2021a
***G. densilamellatus***	**China**	**HMJAU61015**	**ON259034**	**ON259045**	**This study**
*G. densilamellatus*	Republic of Korea	BRNM714927	KP336685	KP336694	Ryoo et al., 2016
*G. dryophilus*	Czech Republic	BRNM695586	JX536143		Antonín et al., 2013
*G. dryophilus*	Germany	BRNM737691	JX536139		Antonín et al., 2013
*G. dryophioides*	Republic of Korea	BRNM781447	MH589967	MH589985	Ryoo et al., 2020
*G. dysodes*	Republic of Korea	BRNM766741	KP336693	KP336701	Ryoo et al., 2016
***G. epiphyllus***	**China**	**HMJAU60990**	**ON259030**	**ON259038**	**This study**
***G. epiphyllus***	**China**	**HMJAU60991**	**ON259029**	**ON259039**	**This study**
*G. erythropus*	Czech Republic	BRNM714784	JX536136		Antonín et al., 2013
*G. erythropus*	USA	JFA12910	DQ449998		Mata et al., 2006
*G. foetidus*	USA	TENN-F-69323	KY026739	KY026739	Petersen and Hughes, 2016
*G. fusipes*	Austria	TENN59300	AF505777		Mata et al., 2006
*G. fusipes*	France	TENN59217	AY256710	AY256710	Mata et al., 2004
*G. graveolens*	France	FF17084	MH422573	MH422572	Unpublished
*G. hakaroa*	New Zealand	PDD87315	KC248410		Cooper and Leonard, 2013
*G. imbricatus*	New Zealand	PDD95489	KC248390		Cooper and Leonard, 2013
*G. impudicus*	USA	BRNM714849	LT594119	LT594119	Ryoo et al., 2016
*G. inusitatus*	Spain	SCMB-4058	JN247553	JN247557	Antonín et al., 2013
*G. iocephalus*	USA	TENN52970	DQ449984	KY019630	Mata et al., 2006
*G. montagnei*	Brazil	URM87715	KT222652		Coimbra et al., 2015
*G. pallipes*	China	GDGM81513	MW582856		Li et al., 2021b
*G. polyphyllus*	USA	TENN59455	AY256695		Ryoo et al., 2016
*G. pygmaeus*	Brazil	URM90003	KX869966	KY088273	Crous et al., 2016
*G. salakensis*	Indonesia	SFSUAWW29	AY263447		Wilson et al., 2004
*G. similis*	China	GDGM78308	MT023352	MT017530	Li et al., 2021a
*G. similis*	Republic of Korea	BRNM766739	KP336692	KP336699	Ryoo et al., 2016
***G. subdensilamellatus***	**China**	**HMJAU60997**	**ON259032**	**ON259042**	**This study**
***G. subdensilamellatus***	**China**	**HMJAU60998**	**ON259033**	**ON259041**	**This study**
***G. subpolyphyllus***	**China**	**HMJAU60999**	**ON259028**	**ON259043**	**This study**
***G. subpolyphyllus***	**China**	**HMJAU61006**	**ON259027**	**ON259044**	**This study**
*G. talisiae*	Brazil	URM87730	KT222655	KX958401	Coimbra et al., 2015
*G. trabzonensis*	Turkey	KATO Fungi 3375	KT271754		Vizzini et al., 2015
*G. variicolor*	Republic of Korea	BRNM714959	LT594121	KP348011	Ryoo et al., 2016
*Mycetinis alliaceus*	Russia	TENN-F-55630	KY696784	KY696752	Petersen and Hughes, 2017
*M. opacus*	USA	TENN-F-59451	KY696755		Petersen and Hughes, 2017
*Paragymnopus foliiphilus*	USA	TENN-F-68183	KY026705	KY026705	Petersen and Hughes, 2016
*P. perforans*	Sweden	TENN-F-50319	KY026625	KY026625	Petersen and Hughes, 2016
*P. pinophilus*	USA	TENN-F-69207	KY026725	KY026725	Petersen and Hughes, 2016
*Rhodocollybia butyracea*	Sweden	TENN53580	AY313293		Mata et al., 2006
*R. maculata*	Dominican Republic	TFB11720	KT205402		Mata et al., 2016

### Data analysis

Based on the BLAST results and morphological similarities, the sequences obtained and related to these samples were collected and are listed in Table 1. A dataset of ITS and nLSU resigns comprised sequences from this study, with 45 representative sequences showing the highest similarity to *Gymnopus* spp. This dataset included all *Gymnopus s. str*. sections (sect. *Androcacei*, sect. *Levipedes*, sect. *Impudicae*, and sect. *Gymnopus*) to explore further the relationships of the newly sequenced Chinese specimens within the genus *Gymnopus*. Moreover, species from allied genera *Rhodocollybia* Singer and *Paragymnopus* J.S. Oliveira were also employed in our phylogenetic analysis. *Mycetinis alliaceus* (Jacq.) Earle ex A.W. Wilson and Desjardin and *Mycetinis opacus* (Berk. and M.A. Curtis) A.W. Wilson & Desjardin were selected as outgroups (Petersen and Hughes, 2016).

Of the dataset, each gene region was aligned using Clustal X (Thonpson, 1997), or MAFFT 7.490 (Katoh and Standley, 2013), and then manually adjusted in BioEdit 7.0.5.3 (Hall, 1999). The datasets first were aligned, and then the ITS and nLSU sequences were combined with Phylosuite v1.2.2 (Zhang et al., 2020). The best-fit evolutionary model was estimated using Modelfinder (Kalyaanamoorthy et al., 2017). Bayesian inference (BI) algorithms were used following the models to perform the phylogenetic analysis. BI was calculated with MrBayes 3.2.6 with a general time-reversible DNA substitution model and a gamma distribution rate variation across the sites (Ronquist and Huelsenbeck, 2003). Four Markov chains were run for two runs from random starting trees for two million generations until the split deviation frequency value was <0.01; the trees were sampled every 100 generations. The first 25% of the sampled trees were discarded as burn-in, while all remaining trees were used to construct a 50% majority consensus tree and calculate the Bayesian posterior probabilities (BPPS). RaxmlGUI 2.0.6 (Edler et al., 2021) was used for maximum likelihood (ML) analysis along with 1,000 bootstraps (BS) replicates using the GTRGAMMA algorithm to perform a tree inference and search for the optimal topology (Vizzini et al., 2015). Then FigTree v1.3.1 was used to visualize the resulting trees.

## Results

### Phylogenetic analysis

In the combined dataset, 65 sequences derived from two gene loci (ITS and nLSU) from 41 samples were used to build phylogenetic trees; 20 were newly generated with 10 ITS sequences and 10 nLSU sequences. Modelfinder selected the best fit model for the combined dataset, and the best fit model for BI is HKY+I+G+F. The phylogenetic construction performed *via* ML and BI analysis for the two combined datasets showed a similar topology.

**Figure 1:**
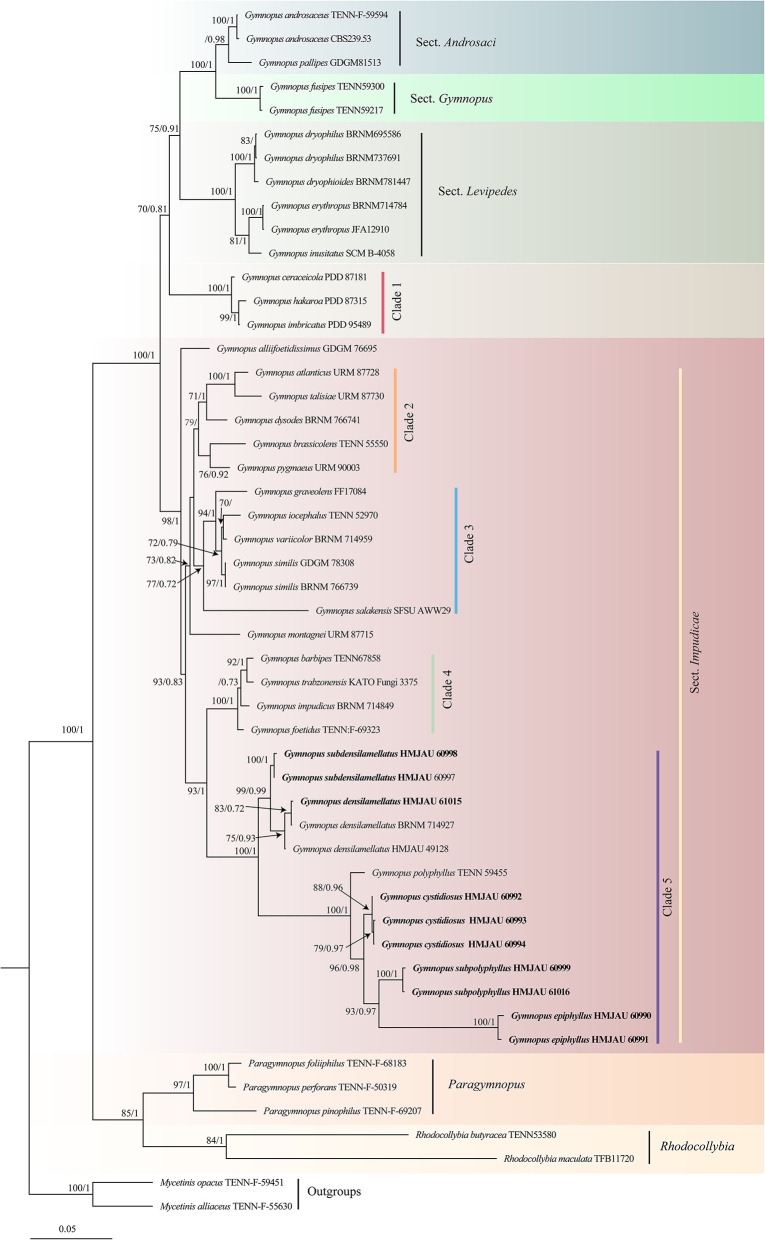
Maximum likelihood phylogenetic tree generated from the ITS and nLSU dataset for the studied *Gymnopus* species and related genera. Two sequences of *Mycetinis* species are selected as outgroups. Bootstrap values (BS) ≥ 70% from ML analysis and Bayesian posterior probabilities (BPPS) ≥ 0.70 are shown on the branches. Newly sequenced collections are indicated in bold.

After trimming, the combined ITS and nLSU dataset represented 39 taxa and 2,407 characters. The Bayesian analysis was run for two million generations and resulted in an average standard deviation of split frequencies of 0.006186. The same dataset and alignment were analyzed using the ML method. Four clades were revealed corresponding to *Gymnopus, Rhodocollybia, Mycetines*, and *Paragymnopus* (Figure 1). Nine sampled specimens formed four new species and were clustered in a clade comprising the species of the *Gymnopus* sect. *Impudicae*. At the same time, one sampled specimen—clustered with *G. densilamellatus* with solid support—was confirmed as a new record for Henan, Jiangxi, and Gansu Province, China.

The phylogeny inferred from ITS and nLSU sequence revealed *Gymnopus* s. str. as a monophyletic genus divided into four clades: sect. *Androcacei* clade, sect. *Levipedes* clade, sect. *Impudicae* clade, and sect. *Gymnopus* clade (Figure 1). The sect. *Impudicae* clade was subsequently divided into four clades. Four new species described in this study and *G. densilamellatus* were gathered into clade 5 with high support.

### Taxonomy

*Gymnopus epiphyllus* J.J. Hu, B. Zhang & Y. Li sp. nov.

**Figure 2:**
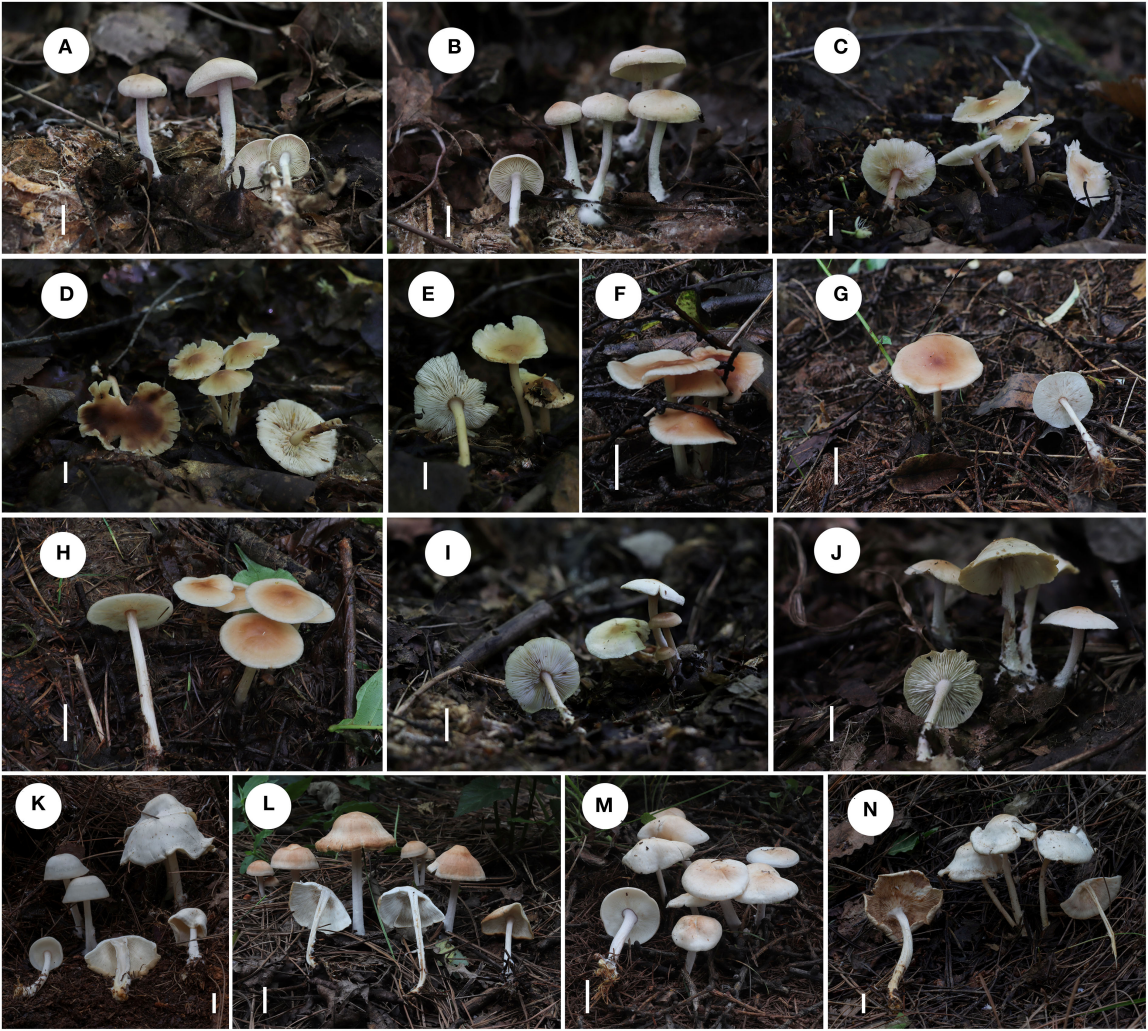
Fresh basidiomata of *Gymnopus* species. **(A,B)** *Gymnopus epiphyllus*; **(C–E)** *Gymnopus cystidiosus*; **(F–H)** *Gymnopus subdensilamellatus*; **(I,J)** *Gymnopus subpolyphyllus*; **(K–N)** *Gymnopus densilamellatus*. Scale bars: 1 cm **(A–N)**.

**Figure 3:**
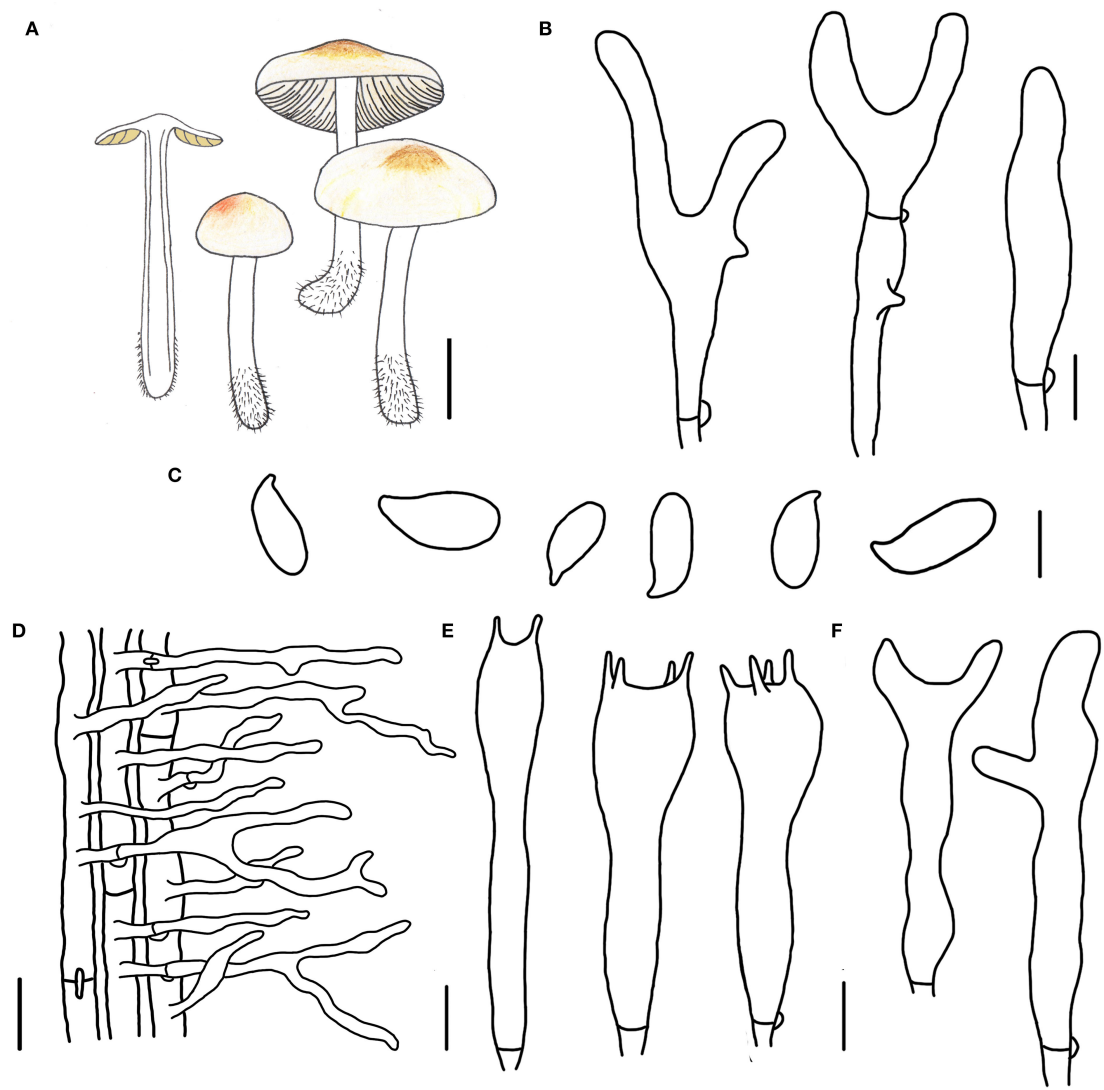
Morphological characteristics of *Gymnopus epiphyllus* (HMJAU 60990, holotype). **(A)** Basidiomata; **(B)** Pileipellis elements; **(C)** Basidospores; **(D)** Caulocysidia; **(E)** Basidia; **(F)** Cheilocystidia; Scale bars: 1 cm **(A)**; 10 μm **(B)**; 5 μm **(C,E,F)**; 20 μm **(D)**.

Figures 2A,B, 3
MycoBank: MB 843736

Etymology: The epithet “*epiphyllus*” means “on leaves,” referring to the species that grows on the decayed leaves in broad-leaved forests.

Diagnosis: This species can be distinguished by its small-sized basidiomata, tomentose pileus with an umbo at disk, smaller basidiospores, and cylindrical terminal elements of pileipellis.

Type. China. Jilin Province, Tonghua City, Ji'an County, Wunvfeng National Forest Park, 13 July 2020, Jia-Jun Hu and Yong-Lan Tuo, HMJAU 60990 (collection no. Hu 556).

Basidiomata small-sized, gregarious. Pileus convex to planate, 1.2–2.2 cm wide, tomentose, nearly white to light brown, with an umbo at disk and light brown to brown; margin nearly white to light brown, entire, involute, covered with dense tomentum. Context thin, fresh, with a strong smell reminding of rotten cabbage or onion. Stipe central, cylindrical, slightly expanded at base, 2.0–3.2 cm long and 0.2–0.3 cm wide, white, covered with pubescence entirely and tomentum at the base, hollow, fibrous. Lamellae adnexed to subfree, light brown, close, unequal.

Basidiospores oblong ellipsoid to cylindrical, 6.0–8.0 × 3.0–3.8(4.0) μm, as the Q = (1.72)1.88–2.30(2.32), Qm = 2.04 ± 0.15, smooth, hyaline, inamyloid, thin-walled. Basidia clavate, (13)17–31(32) × 4–6(7) μm, two or four spored, thin-walled, hyaline. Cheilocystidia irregularly clavate, with a mucro becoming elongated filiform with age, or with a projection at apex, 15–32(37) × 3–5 μm, thin-walled, hyaline. Caulocystidia numerous, clavate or irregularly clavate to branched, 40–65(75) × (3)4–6 μm, thin-walled, hyaline. Pileipellis a cuits; hyphal elements branched, hyaline, (3)5–12(13) μm wide; clamp connections abundant.

Habitat. Saprophytic on fallen leaves in broad-leaved forests.

Other specimens examined. China. Jilin Province, Tonghua City, Ji'an County, Wunvfeng National Forest Park, 13 July 2020, Jia-Jun Hu and Yong-Lan Tuo, HNJAU 60991 (collection no. Hu 560).

Note. *Gymnopus epiphyllus* is characterized by small-sized basidomata with a yellowish-brown umbo and tomentose pileus, light khaki lamellae, white and tomentose stipe, and small basidiospores. Morphologically, *G. epiphyllus* is similar to *Gymnopus atlanticus* V. Coimbra, Pinheiro, Wartchow and Gibertoni with pale brown pileus and light khaki lamellae. However, *G. epiphyllus* differs from *G. atlanticus* in a tomentose pileus with a brown umbo at disk, nearly white and tomentose stipe, not pyriform, lageniform to somewhat sphaeropedunculate, versiform, or moniliform rostrum terminal elements of pileipellis (Coimbra et al., 2015), and smaller basidiospores.

*Gymnopus cystidiosus* J.J. Hu, B. Zhang & Y. Li sp. nov.

**Figure 4:**
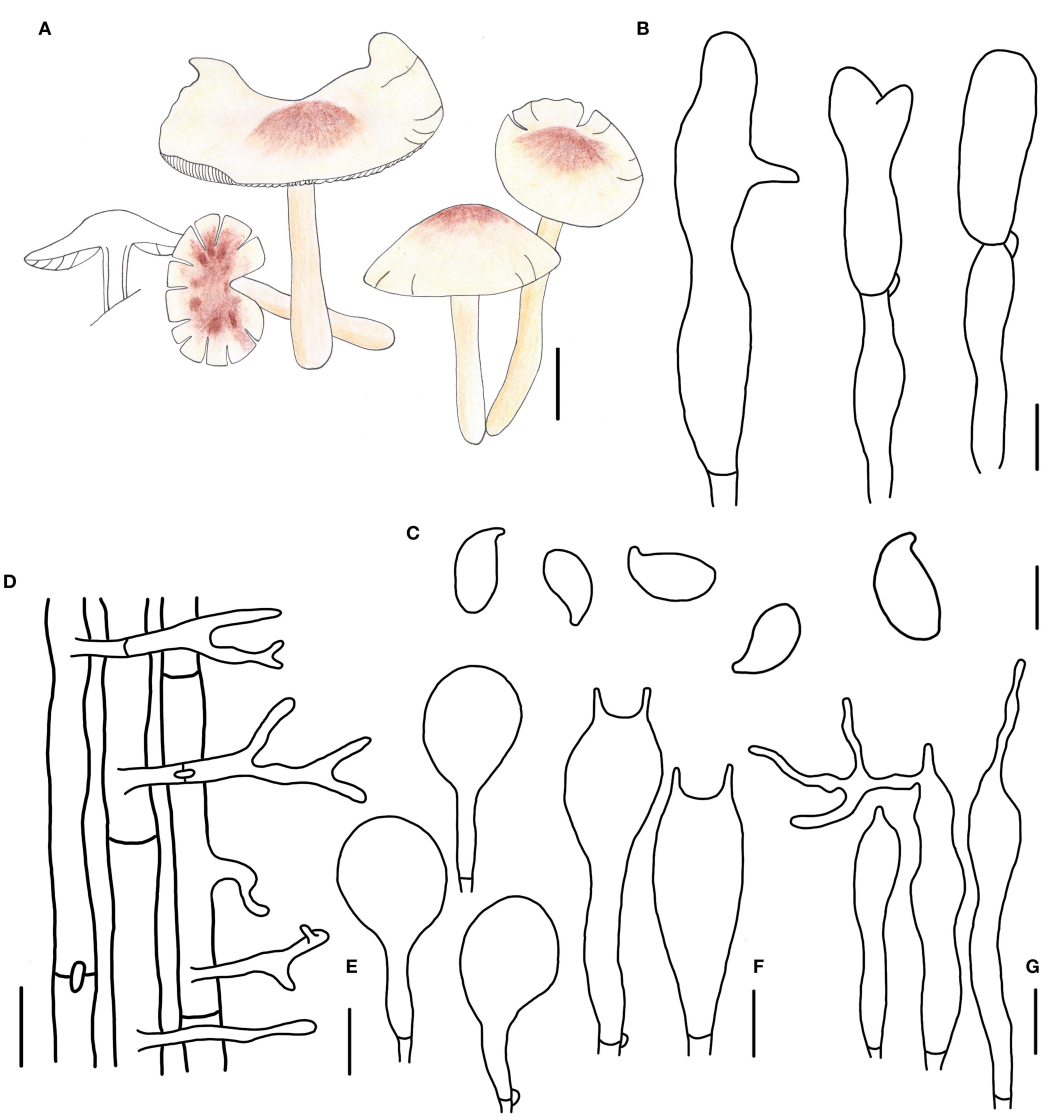
Morphological characteristics of *Gymnopus cystidiosus* (HMJAU 60992, holotype). **(A)** Basidiomata; **(B)** Pileipellis elements; **(C)** Basidospores; **(D)** Caulocystidia; **(E)** Pleurocystidia; **(F)** Basidia; **(G)** Cheilocystidia; Scale bars: 1 cm **(A)**; 10 μm **(B,E)**; 20 μm **(D)**; 5 μm **(C,F,G)**.

Figures 2C–E, 4
MycoBank: MB 843738
Etymology: The epithet “*cystidiosus*” refers to the species having pleurocystidia.
Diagnosis: This species can be distinguished from other species by its small basidiomata, glabrous pileus with a dark brown umbo, close and white lamellae, presence of pleurocystidia, and smaller basidiospores.
Type. China. Jilin Province, Tonghua City, Ji'an County, Wunvfeng National Forest Park, 21 July 2020, Jia-Jun Hu and Yong-Lan Tuo, HMJAU 60992 (collection no. Hu 577).

Basidiomata small-sized, solitary to gregarious. Pileus convex to applanate, or reflex, 1.8–3.6 cm wide, glabrous, hygrophanous, brown to dark brown at the disc, yellow to light brown outwards; margin nearly white to light yellow then becoming light brown, straight at first then becoming reflex. Context thin, fresh, with a strong smell reminding of rotten cabbage or onion. Stipe central, cylindrical, tapering downwards sometimes, 2.4–4.5 cm long and 0.2–0.4 cm wide, yellow or fresh-colored to pink-fresh, covered with pubescence entirely, tomentose at the base, hollow, fibrous. Lamellae adnexed to free, white, becoming dark brown when old, close, unequal.

Basidiospores ellipsoid to oblong ellipsoid, (5.1)5.2–6.4(7.0) × (2.8)3.0–4.0 μm, as the Q = (1.50)1.55–2.00(2.14), Qm = 1.79 ± 0.16, smooth, hyaline, inamyloid, thin-walled. Basidia clavate, 18–28 × 3–7 μm, usually 2-spored, occasionally 4-spored, thin-walled, hyaline. Cheilocystidia irregularly clavate, with a mucro that becomes elongated filiform with age, or projections like at apex, (15)19–32(34) × 3–5 μm, thin-walled, hyaline. Pleurocystidia scattered, pyriform to broadly fusoid-ventricose, 20–32 × (5)8–12(13) μm, thin-walled, hyaline. Caulocystidia clavate to branched, (15)27–50 × 3–5 μm, thin-walled, hyaline. Pileipellis a cuits, branched, hyaline, 7–15 μm wide; clamp connections abundant.

Habitat. Saprophytic on fallen leaves in broad-leaved forests.

Other specimens examined. China. Jilin Province, Tonghua City, Ji'an County, Wunvfeng National Forest Park, 21 July 2020, Jia-Jun Hu and Yong-Lan Tuo, HMJAU 60993 (collection no. Hu 578); Tonghua City, Ji'an County, Wunvfeng National Forest Park, 21 July 2020, Jia-Jun Hu and Yong-Lan Tuo, HMJAU 60994 (collection no. Hu 579); Tonghua City, Ji'an County, Wunvfeng National Forest Park, 21 July 2020, Jia-Jun Hu and Yong-Lan Tuo, HMJAU 60995 (collection no. Hu 581); Tonghua City, Ji'an County, Wunvfeng National Forest Park, 5 August 2020, Jia-Jun Hu and Yong-Lan Tuo, HMJAU 60996 (collection no. Hu 644).

Note. *Gymnopus cystidiosus* is characterized by its small basidiomata, glabrous pileus with a dark brown umbo, dark brown spots when old, crowded, and nearly white lamellae, fresh to pink and glabrous stipe, presence of pleurocystidia, and smaller basidiospores. *Gymnopus cystidiosus* is closely related to *G. epiphyllus* in morphology and phylogeny because of the pileus with an umbo. However, *G. cystidiosus* differs from *G. epiphyllus* by its glabrous pileus with brown spots when old, white and crowded lamellae, glabrous and fresh to pink stipe, presence of pleurocystidia, projection cheilocystidia, and smaller basidiospores.

*Gymnopus subdensilamellatus* J.J. Hu, Y.L. Tuo, B. Zhang & Y. Li sp. nov.

**Figure 5:**
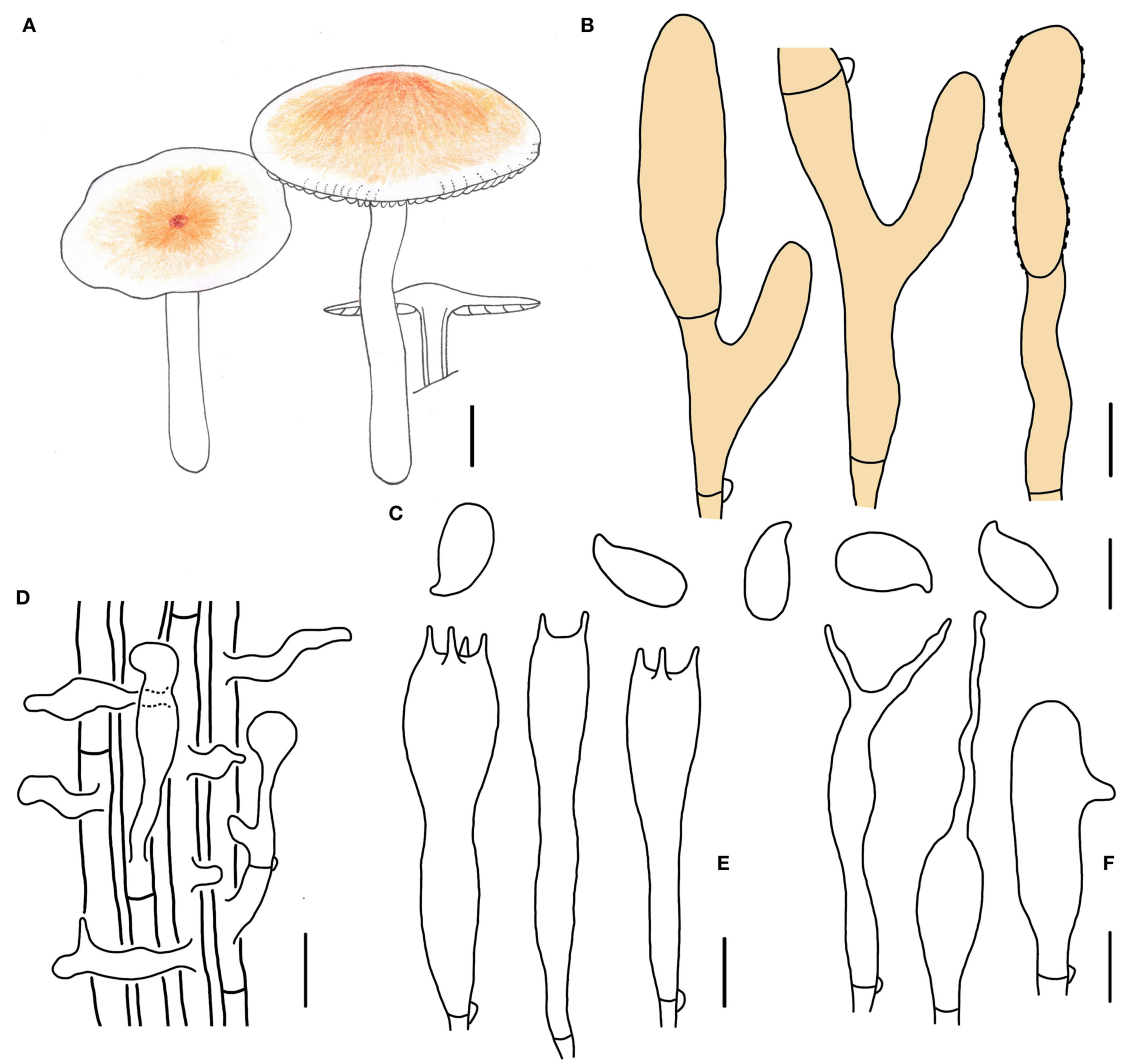
Morphological characteristics of *Gymnopus subdensilamellatus* (HMJAU 60997, holotype). **(A)** Basiaiomata; **(B)** Pileipellis elements; **(C)** Basidospores; **(D)** Caulocystidia; **(E)** Basidia; **(F)** Cheilocystidia; Scale bars: 1 cm **(A)**; 10 μm **(B)**; 20 μm **(D)**; 5 μm **(C,E,F)**.

Figures 2F–H, 5
MycoBank: MB 843743
Etymology: sub = near; the epithet “*subdensilamellatus*” refers to this species closely related to *G. densilamelatus*.
Diagnosis: This species is differentiated from others by its brown, and none spotted pileus, crowded lamellae, striate stipe, and small basidiospores and Qm.
Type. China. Jilin Province, Tonghua City, Ji'an County, Wunvfeng National Forest Park, 4 September 2020, Yong-Lan Tuo, HMJAU 60997 (collection no. Hu 675).

Basidiomata small- to medium-sized, gregarious. Pileus convex to applanate, 2.5–5.0 cm wide, glabrous, dark brown and slightly depressed at the center, pale colored outwards at first, yellow to brown, dark brown when mature; margin nearly white to light yellow, entire, involute, wavy sometimes. Context thin, fresh, with a strong smell reminding of rotten cabbage or onion. Stipe central, cylindrical to clavate, 5.0–7.2 cm long and 0.2–0.7 cm wide, nearly white to dirty white, almost white to light reddish brown at apex, pruinose, covered with white tomentose at the base, with longitudinal striate, hollow, fibrous. Lamellae adnexed to subfree, white, extremely close, unequal.

Basidiospores oblong ellipsoid to cylindrical, 6.0–7.2(7.4) × 3.0–3.8(4.0) μm, as the Q = (1.75)1.82–2.07(2.19), Qm = 1.95 ± 0.10, smooth, hyaline, inamyloid, thin-walled. Basidia clavate, (16)17–28(30) × 4–6 μm, two to four spored, thin-walled, hyaline. Cheilocystidia irregularly clavate, with a mucro that becomes elongated filiform with age, or with projections sometimes, 20–28(30) × 4–7 μm, thin-walled, hyaline. Caulocystidia numerous, irregularly clavate, branched, (12)15–50 × 4–10(11) μm, thin-walled, hyaline. Pileipellis a cuits, branched, brown, 6–7(13) μm wide; clamp connections abundant.

Habitat. Saprophytic on fallen leaves in coniferous forest.

Other specimen examined. China. Jilin Province, Tonghua City, Ji'an County, Wunvfeng National Forest Park, 4 August 2020, Yong-Lan Tuo, HMJAU 60998 (collection no. Hu 667).

Note. *Gymnopus subdensilamellatus* is characterized by its unchangeable brown pileus with white to light brown margin, crowded lamellae, white stipe with light reddish brown at apex, and smaller Qm. *Gymnopus subdensilamellatus* differs from *G. densilamellatus* by its unchangeable brown pileus (*G. densilamellatus* have a brown then white pileus with an ochraceous brownish center; Ryoo et al., 2016), an irregularly clavate caulocystidia, and a mucro to projections cheilocystidia.

*Gymnopus subpolyphyllus* J.J. Hu, B. Zhang & Y. Li sp. nov.

**Figure 6:**
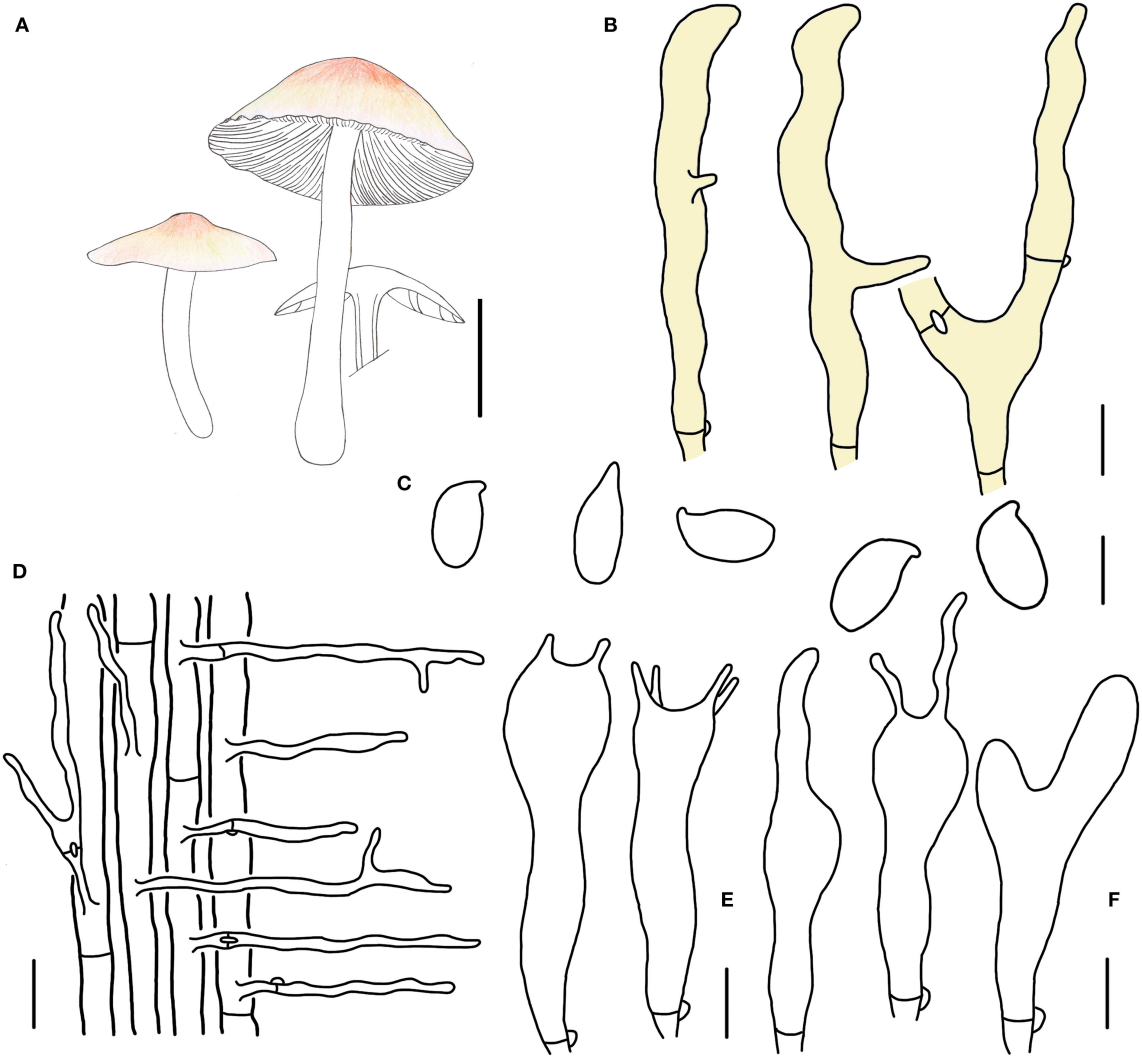
Morphological characteristics of *Gymnopus subpolyphyllus* (HMJAU 60999, holotype). **(A)** Basidiomata; **(B)** Pileipellis elements; **(C)** Basidospores; **(D)** Caulocystidia; **(E)** Basidia; **(F)** Cheilocystidia; Scale bars: 1 cm **(A)**; 10 μm **(B)**; 5 μm **(C,E,F)**; 20 μm **(D)**.

Figures 2I, J, 6
MycoBank: MB 843744
Etymology: sub = near; the epithet “*subpolyphyllus*” refers to this species closely related to *G. polyphyllus*.
Diagnosis: This species can be distinguished from other species by its small basidiomata, pileus with an umbo, tomentose margin and stipe, branched or projected cheilocystidia, branched and long caulocystidia, and small basidiospores.
Type. China. Jilin Province, Tonghua City, Ji'an County, Wunvfeng National Forest Park, 13 July 2020, Jia-Jun Hu and Yong-Lan Tuo, HMJAU 60999 (collection no. Hu 561).

Basidiomata small-sized, gregarious. Pileus plano-hemispherical to convex, 1.4–2.3 cm wide, glabrous, with an umbo sometimes, hygrophanous and pinkish brown when young, then brown to fresh-pink at the disc, pale colored outwards; margin pinkish brown when young, then nearly white to light yellow, involute at first, then becoming straight, reflex sometimes, tomentum. Context thin, fresh, with a strong smell reminding of rotten cabbage or onion. Stipe central, cylindrical, 2.2–3.0 cm long and 0.2–0.3 cm wide, pinkish brown when young, then pale reddish brown, glabrous at the upper part at first, then entirely tomentose, hollow, fibrous. Lamellae adnexed, cream, close, unequal.

Basidiospores ellipsoid to cylindrical, (5.1)5.2–7.0 × (2.9)3.0–4.0 μm, as the Q = (1.48)1.58–2.19(2.33), Qm = 1.84 ± 0.20, smooth, hyaline, inamyloid, thin-walled. Basidia clavate, 17–27 × 4–6 μm, two or four spored, thin-walled, hyaline. Cheilocystidia irregularly clavate, with a mucro that becomes elongated filiform with age, or with projections sometimes, 18–29(30) × 3–6(7) μm, thin-walled, hyaline. Caulocystidia abundant, long clavate, branched, 40–90(100) × 3–6 μm, thin-walled, hyaline. Pileipellis a cuits; hyphal elements branched, hyaline to light yellow, (5)6–10(11) μm wide; clamp connections abundant.

Habitat. Saprophytic on fallen leaves in broad-leaved forests.

Other specimens examined. China. Jilin Province, Tonghua City, Ji'an County, Wunvfeng National Forest Park, 17 July 2020, Jia-Jun Hu and Yong-Lan Tuo, HMJAU 61016 (collection no. Hu 566); Tonghua City, Ji'an County, Wunvfeng National Forest Park, 17 July 2020, Jia-Jun Hu and Yong-Lan Tuo, HMJAU 61000 (collection no. Hu 572).

Note. *Gymnopus subpolyphyllus* is characterized by its small basidiomata, glabrous pileus with a light brown umbo occasionally, tomentose margin and stipe, cheilocystidia branched or projection at apex, abundant, branched and long caulocystidia, and small basidiospores. *Gymnopus subpolyphyllus* is closely related to *G. polyphyllus* due to its similar appearance. However, *G. subpolyphyllus* differs from *G. polyphyllus* by its glabrous pileus, cream lamellae, tomentose stipe, different shape of cheilocystidia, and slightly wider basidiospores (Halling, 1983).

*Gymnopus densilamellatus* Antonín, Ryoo & Ka

Figures 2K–N, 7

**Figure 7:**
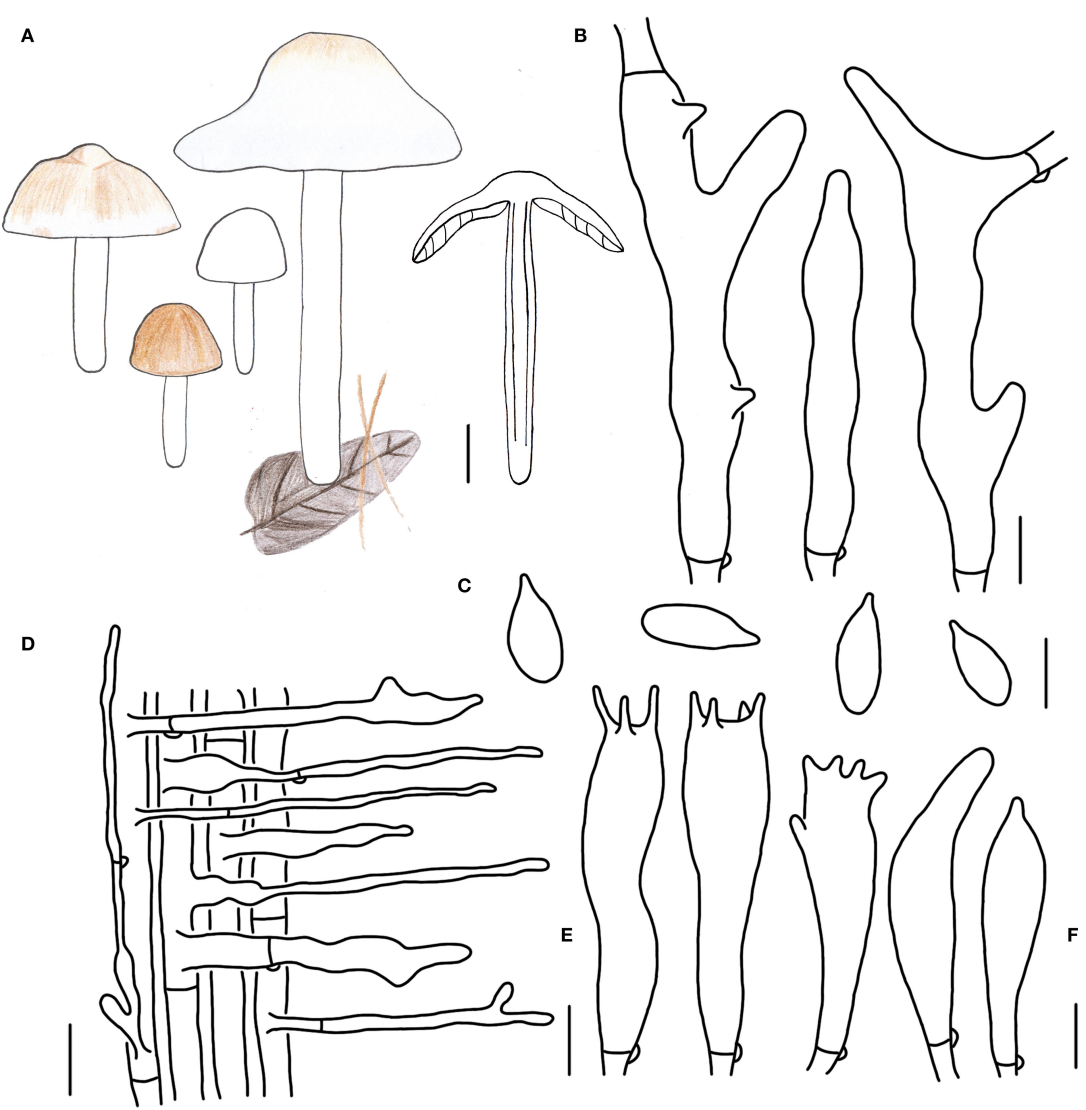
Morphological characteristics of *Gymnopus densilamellatus* (HMJAU 61015). **(A)** Basidiomata; **(B)** Pileipellis elements; **(C)** Basidospores; **(D)** Caulocystidia; **(E)** Basidia; **(F)** Cheilocystidia; Scale bars: 2 cm **(A)**; 10 μm **(B)**; 5 μm **(C,E,F)**; 20 μm **(D)**.

Basidiomata medium- to large-sized, usually gregarious. Pileus hemispherical or plano-hemispherical when young, then becoming convex, applanate when mature, 2.0–7.3 cm wide, usually brown, or white sometimes when young, then paler, fading to light brown or yellowish-brown at the center, whitish outwards, becoming white with a light brown center when mature, glabrous, hygrophanous; margin entire, inflexed then straight, white, or brown at first then fading to white when mature. Context thin, white, fresh, with a strong smell reminding of rotten cabbage or onion. Stipe central, cylindrical, 2.2–10.3 cm long and 0.2–0.3 cm wide, usually white, with brown tone sometimes, glabrous, tomentose sometimes, hollow, fibrous. Lamellae free, white to yellowish-white, brown when old, close to crowded, unequal.

Basidiospores oblong ellipsoid to cylindrical, (5.2)5.6–8.0 × 2.8–4.0 μm, as the Q = (1.78)1.80–2.35(2.67), Qm = 2.11 ± 0.23, smooth, hyaline, inamyloid, thin-walled. Basidia clavate, (17)20–25 × 4–6 μm, two or four spored, thin-walled, hyaline. Cheilocystidia irregularly clavate, with projections, 17–23 × 3–6 μm, thin-walled, hyaline. Caulocystidia abundant, long clavate, branched, 30–170 × 3–8 μm, thin-walled, hyaline. Pileipellis a cuits; hyphal elements branched, hyaline to brown, (6)7–13(15) μm wide; clamp connections abundant.

Habitat. Saprophytic on fallen leaves in coniferous and broad-leaved mixed forest.

Specimens examined. China. Jilin province, Baishan City, Fusong County, Yongqing Forest Farm, 7 July 2018, Jia-Jun Hu and Ao Ma, HMJAU 61002 (collection no. Hu 31); Baishan City, Fusong County, Beigang Town, 22 August 2021, Jia-Jun Hu, Gui-Ping Zhao, and Bo Zhang, HMJAU 61003 (collection no. Hu 881); Changchun City, Jingyuetan National Forest Park, 15 July 2018, Jia-Jun Hu, HMJAU 61004 (collection no. Hu 49), HMJAU 61005 (collection no. Hu 50); 31 August 2018, Jia-Jun Hu, HMJAU 61006 (collection no. Hu 72); Jilin City, Jiaohe county, Lafa mountain National Forest Park, 9 September 2018, Jia-Jun Hu and Bo Zhang, HMJAU 61007 (collection no. Hu 102); 19 August 2021, Jia-Jun Hu and Bo Zhang, HMJAU 61008 (collection no. Hu 866); Jilin City, Jiaohe county, Zhuque mountain National Forest Park, 18 August 2021, Jia-Jun Hu and Bo Zhang, HMJAU 61009 (collection no. Hu 841), HMJAU 61010 (collection no. Hu 845); Tonghua City, Ji'an County, Wunvfeng National Forest Park, 21 August 2021, Yong-Lan Tuo, HMJAU 61011 (collection no. Hu 361), HMJAU 61012 (collection no. Hu 363); Jiangxi Province, Ji'an City, Jinggangshan County, Jinggang Mountain, 19 July 2019, Bo Zhang, HMJAU 61013 (collection no. Hu 211); Gansu Province, Zhangye City, Xishui Nature Reserve Station, 14 August 2019, Jia-Jun Hu, Wang Yang, and Zhi-Hui Luo, HMJAU 61014 (collection no. Hu 258); Henan Province, Zhumadian City, Biyang County, Baiyun Mountain, 10 July 2021, Jia-Jun Hu, Gui-Ping Zhao, and Bo Zhang, HMJAU 61015 (collection no. Hu 772).

Note. *Gymnopus densilamellatus* was initially described in South Korea; then Li et al. (2021a) first recorded it in China in 2021. More than five specimens of *G. densilamellatus* were collected from Henan, Jiangxi, and Gansu Province, China.

There are some differences between the specimens we collected and the original descriptions. According to the original description, the pileus is brown to reddish-brown at first, then becoming paler, brownish orange to brown at the center and whitish outwards, later whitish, with an ochraceous brownish center when mature (Ryoo et al., 2016). While during our investigation, some white basidiomata specimens from young to mature were found, and the morphological characters were carefully examined.

### Key to the reported species of *Gymnopus* sect. *Impudicae*

1 Basidiomata typically collybioid or marasmioid............................ 2
1 Basidiomata not typically collybioid or marasmioid .........................
...................................................................... *G. montagnei*
2 Cheilocystidia absence .................................................. 3
2 Cheilocystidia presence ................................................. 5
3 Caulocystidia presence .................................................. 4
3 Caulocystidia absence ................................................. *G. imbracatus*
4 Pileus and stipe are concolor, uniform color of stipe
...................................................................... *G. ceraceicola*
4 Pileus and stipe heterochromatic, light color at the base of stipe ............ *G. hakaroa*
5 Pileus sulcate, lamellae adnexed to adnate, light yellow to brow ............. 6
5 Pileus without sulcate or with inconspicuous sulcate ...................... 10
6 Pileipellis Rameales structues........................................... *G. atlanticus*
6 Pileipellis a cuits........................................................7
7 Basidiomata are light colored—yellow to yellowish brown.................. 8
7 Basidiomata are dark colored—reddish brown to dark brown
....................................................................... 9
8 Basidiomata grows on fallen leaves or ground ............................ *G. talisiae*
8 Basidiomata grows on rotten wood ...................................... *G. pygmaeus*
9 Basidiospores shorter than 7.5 μm *G. brassicolens*
9 Basidiospores longer than 7.5 μm *G. dysodes*
10 Lamellae moderately distant, cheilocystidia projection(s) at apex, and branched pileipellis elements .................................................... 11
10 Lamellae closed to crowded, cheilocystidia clavate or projection(s) at apex ..12
11 Lamellae yellow to brown .............................................. *G. similis*
11 Lamellae brownish orange, sometimes with a light violaceous tinge......... *G. variicolor*
12 Basidiomata small-sized, with a striped pileus ............................ 13
12 Basidiomata usually medium- to large-sized, with a glabrous pileus......... 15
13 Stipe mostly lilac or pinkish tinged with a dark colored base
...................................................................... *G. impudicus*
13 Stipe reddish brown.................................................... 14
14 Basidiomata marasmioid ............................................... *G. foetidus*
14 Basidiomata collybioid................................................. *G. trabzonensis*
15 Pleurocystidia present.................................................. *G. cystidiatus*
15 Pleurocystidia absent .................................................. 16
16 Pileus covered with tomentose .......................................... 17
16 Pileus glabrous ....................................................... 18
17 Pileipellis hyaline in 3% KOH, basidiospores longer than 7 μm *G. epiphyllus*
17 Pileipellis light yellow in 3% KOH, basidiospores shorter than 7 μm *G. subpolyphyllus*
10 Color of pileus unchangeable, branched, or a mucro to projections cheilocystidia, basidiospores shorter than 7.2 μm
...................................................................... *G. subdensilamellatus*
18 Color of pileus in most species changeable from brown to nearly white, irregularly clavate, or finger-like cheilocystidia, basidiospores longer than 7.2 μm *G. densilamellatus*

## Discussion

This study describes four new species belonging to *Gymnopus* from Northeast China. They are well-supported by molecular phylogenetic and morphological evidence. Our newly recognized and delimited species are occurring in broad-leaved or coniferous forests.

The phylogenetic studies on sect. *Impudicae* were started in recent 20 years and with poor focus. Wilson and Desjardin (2005) and Mata et al. (2006) indicated that subsect. *Impudicae* should be an independent section, not a subsection of sect. *Vestipedes*, although they shared a tomentose stipe and pileipellis as a simple cutis (Antonín and Noordeloos, 1993, 1997). Later, Antonín and Noordeloos (2010) revised the limits of this group and raised it to a sectional rank. Coimbra et al. (2015) studied on sect. *Impudicae* from Northern Brazil, based on ITS sequence, concluded that the species of sect. *Impudicae* were mainly divided into two subclades—subclade *impudici* and subclade *foetidi*. The diagnostic features of the section proposed by Antonín and Noordeloos (2010) do not reflect all the morphological variability of members of the sect. *Impudicae*.

Our combined phylogenetic result differs from Coimbra et al. (2015), while similar to Li et al. (2021a). In our phylogenetic analysis, sect. *Impudicae* consisted of four significant subclades: clade 2, clade 3, clade 4, and clade 5. Clade 2 includes five species—*Gymnopus atlanticus* V. Coimbra, Pinheiro, Wartchow and Gibertoni, *Gymnopus brassicolens* (Romagn.) Antonín and Noordel., *Gymnopus dysodes* (Halling) Halling, *Gymnopus pygmaeus* V. Coimbra, E. Larss., Wartchow and Gibertoni, and *Gymnopus talisiae* V. Coimbra, Pinheiro, Wartchow and Gibertoni—are characterized by pileus sulcate, lamellae adnexed to adnate, light yellow to brown. Clade 3 consists of *Gymnopus graveolens* (Pers.) Gray, *Gymnopus iocephalus* (Berk. and M.A. Curtis) Halling, *Gymnopus similis* Antonín, Ryoo and Ka, *Gymnopus salakensis* A.W. Wilson, Desjardin and E. Horak, and *Gymnopus variicolor* Antonín, Ryoo, Ka and Tomšovský. Clade 3 is characterized by the moderately distant lamellae, cheilocystidia projection(s) at the apex, and branched pileipellis elements. *Gymnopus barbipes* R.H. Petersen and K.W. Hughes, *Gymnopus foetidus* (Sowerby) J.L. Mata and R.H. Petersen, *Gymnopus impudicus* (Fr.) Antonín, Halling and Noordel., and *Gymnopus trabzonensis* Vizzini, Antonín, Sesli and Contu clustered into one branch, forming clade 4. Clade 4 is characterized by small-sized basidiomata with brown tones and striate pileus, clavate cheilocystidia, and sometimes rostrate or projection(s) at apex, 4-spored basidia. *Gymnopus densilamellatus, G. polyphyllus*, and our four new species form clade 5. All the species included in clade 5 are featured by the yellow to brown pileus disk, close to crowded lamellae, basidiospores 5–8 μm long, cheilocystidia with projection(s) at apex, and branched pileipellis elements.

However, two species—*Gymnopus alliifoetidissimus* T.H. Li and J.P. Li and *Gymnopus montagnei* (Berk.) Redhead form two independent clades respectively. These two species with strong smell were not morphologically typical species of sect. *Impudicae*. Besides, *G. montagnei* lacks lamellae. In addition, *Gymnopus ceraceicola* J.A. Cooper and P. Leonard, *Gymnopus hakaroa* J.A. Cooper and P. Leonard, and *Gymnopus imbricatus* J.A. Cooper and P. Leonard, described in New Zealand (clade 1), initially considered as the members of the sect. *Impudicae*, formed an independent clade close to the sect. *Androsacei*, sect. *Gymnopus*, and sect. *Levipedes*, far away from sect. *Impudicae*, which was different from Li et al. (2021a). The key features of these three species are small-sized and marasmioid basidiomata, growing on rotten wood, lacking of cheilocystidia and pleurocystidia, and presence of gelatinized pileipellis; in contrast, the species of sect. *Impudicae* are usually collybioid, presence of cheilocystidia. Furthermore, this section includes some species without distinct smell—*G. atlanticus, Gymnopus barbipes*, and *G. salakensis*. Thus, the morphological limits of sect. *Impudicae* needs to be reevaluated, and relationships within this section or among genus *Gymnopus* need to be further clarified.

## Data availability statement

The datasets presented in this study can be found in online repositories. The names of the repository/repositories and accession number(s) can be found in the article/supplementary material.

## Author contributions

YL and BZ: conceptualization and supervision. YL, BZ, L-RS, and J-JH: experimental design and methodology. J-JH and G-PZ: performance of practical work. J-JH, Y-LT, and LY: statistical analyzes. J-JH and BZ: validation. J-JH: writing—original draft preparation. BZ: writing—review and editing, project administration, and funding acquisition. All authors have read and agreed to the published version of the manuscript.

## Funding

This study is funded by the Natural Science Foundation of China (Nos. 31970020 and 31860582), National Key R & D of the Ministry of Science and Technology (2019YFD1001905-33), Research on the Creation of Excellent Edible Mushroom Resources and High Quality & Efficient Ecological Cultivation Technology in Jiangxi Province (20212BBF61002), Modern Agricultural Scientific Research Collaborative Innovation Special Project (JXXTCXBSJJ202212), and 111 program (No. D17014).

## Conflict of interest

The authors declare that the research was conducted in the absence of any commercial or financial relationships that could be construed as a potential conflict of interest.

## Publisher's note

All claims expressed in this article are solely those of the authors and do not necessarily represent those of their affiliated organizations, or those of the publisher, the editors and the reviewers. Any product that may be evaluated in this article, or claim that may be made by its manufacturer, is not guaranteed or endorsed by the publisher.
